# Adaptive radiation of chemosymbiotic deep-sea mussels

**DOI:** 10.1098/rspb.2013.1243

**Published:** 2013-11-07

**Authors:** Julien Lorion, Steffen Kiel, Baptiste Faure, Masaru Kawato, Simon Y. W. Ho, Bruce Marshall, Shinji Tsuchida, Jun-Ichi Miyazaki, Yoshihiro Fujiwara

**Affiliations:** 1 Marine Ecosystems Research Department, Japan Agency for Marine-Earth Science and Technology (JAMSTEC), 2-15 Natsushima, Yokosuka 237-0061, Japan; 2 Geoscience Center, Geobiology Group, University of Göttingen, Goldschmidtstrasse 3, Göttingen 37077, Germany; 3 Department of Biology, Pennsylvania State University, University Park, PA 16802, USA; 4 School of Biological Sciences, University of Sydney, Sydney, New South Wales 2006, Australia; 5 Museum of New Zealand Te Papa Tongarewa, PO Box 467, 169 Tory St., Te Aro, Wellington 6011, New Zealand; 6 Faculty of Education and Human Sciences, University of Yamanashi, Kofu, Yamanashi 400-8510, Japan

**Keywords:** phylogenetics, adaptive radiation, symbiosis, hydrothermal vent, cold seep, organic falls

## Abstract

Adaptive radiations present fascinating opportunities for studying the evolutionary process. Most cases come from isolated lakes or islands, where unoccupied ecological space is filled through novel adaptations. Here, we describe an unusual example of an adaptive radiation: symbiotic mussels that colonized island-like chemosynthetic environments such as hydrothermal vents, cold seeps and sunken organic substrates on the vast deep-sea floor. Our time-calibrated molecular phylogeny suggests that the group originated and acquired sulfur-oxidizing symbionts in the Late Cretaceous, possibly while inhabiting organic substrates and long before its major radiation in the Middle Eocene to Early Oligocene. The first appearance of intracellular and methanotrophic symbionts was detected only after this major radiation. Thus, contrary to expectations, the major radiation may have not been triggered by the evolution of novel types of symbioses. We hypothesize that environmental factors, such as increased habitat availability and/or increased dispersal capabilities, sparked the radiation. Intracellular and methanotrophic symbionts were acquired in several independent lineages and marked the onset of a second wave of diversification at vents and seeps. Changes in habitat type resulted in adaptive trends in shell lengths (related to the availability of space and energy, and physiological trade-offs) and in the successive colonization of greater water depths.

## 1. Introduction

Adaptive radiation is broadly defined as the rapid diversification of species and of their adaptations to the environment in response to natural selection and ecological opportunities [1]. The radiations of Darwin's finches and African Rift Lake cichlids are well known and have become popular beyond academic research, but these examples may just be the ‘tip of an evolutionary iceberg’, because adaptive radiations can take place on a broad range of time scales and taxonomic levels, and may even include the Cambrian explosion of life [2]. The fauna living at hydrothermal vents and cold seeps in the deep sea represents another remarkable, yet poorly understood case of radiation. These habitats are characterized by extreme physico-chemical features and a scarcity of primary photosynthetic production, and the animals living there thrive because of their symbiotic relationships with bacteria able to use sulfide and/or methane as energy sources [3]. Initially considered as archaic faunas that survived major extinction events [4], molecular age estimates and fossil records suggested that most of the modern vent and seep animals appeared during a short time interval between the Late Mesozoic and the Early Cenozoic [5,6].

Mussels of the bivalve family Mytilidae are particularly suitable as model organisms to study the roles of ecological opportunities, symbioses and other adaptations in the evolution of deep-sea chemosymbiotic faunas. Indeed, they dominate many vents and seeps, but are also common in other sulfide-rich habitats such as whale carcasses and sunken wood [7], which are considered to be evolutionary stepping stones to deep-sea vents [8]. In addition to this diversity of habitats, deep-sea mussels have a remarkable range of symbiotic types, including intracellular and extracellular symbionts, and the ability to host either or both sulfur-oxidizing and methanotrophic symbionts [7].

We investigated the evolutionary history of deep-sea symbiotic mussels by estimating phylogenetic relationships from a comprehensive dataset including all known lineages and 80% of the known species, collected from virtually all ocean basins. In an analysis of five gene fragments, including mitochondrial and nuclear DNA, calibrated with three reliable fossils, we (i) characterized speciation rates through time; (ii) reconstructed the evolution of habitat use (environmental type and depth), body size, symbiont type (sulfur and/or methane oxidizer) and the degree of the physiological integration of the symbiont with the host (intracellular versus extracellular symbioses); and (iii) evaluated the impact of these biological and ecological factors on speciation rates, and their respective roles in the evolution of deep-sea symbiotic mussels.

## 2. Material and methods

### (a) Sampling

Fourteen species of vent and seep mussels from the western Pacific were sampled using the manned submersible Shinkai 6500 and remotely operated Hyper Dolphin vehicle (see electronic supplementary material, table S1). From experimental bone deployments in Japanese waters, we collected the three undescribed species *Idas* sp SAL4, *Idas* evolutionary significant unit (ESU) D and *Idas* ESU R [9]. Upon recovery, pieces of the gills of *Bathymodiolius aduloides* and the three undescribed species were fixed with 2.5% glutaraldehyde in filtered seawater for 24 h and preserved in filtered seawater with 10 mM sodium azide at 4°C. Remaining tissues and other specimens were fixed in 99% ethanol for DNA analysis. We also analysed alcohol-fixed foot tissues of 15 species associated with sunken organic substrates collected in the western Pacific during cruises of the Tropical Deep-sea Benthos program [9,10]. Finally, we included alcohol-fixed specimens from the Atlantic Ocean and Mediterranean Sea, including two species from organic falls, six species from hydrothermal vents, five species from cold seeps and the subtidal outgroup species *Modiolus modiolus* (Linnaeus, 1758).

### (b) Molecular analyses

Template DNA from feet and gills were extracted to analyse host and symbiont genes, respectively, using the QIAamp DNA Micro Kit (Qiagen, USA). A fragment of the small subunit 16S rRNA was amplified to characterize symbionts in 10 species that had not been studied previously (see electronic supplementary material, table S1). Fragments of mitochondrial COI, NADH4 and 16S, and nuclear 28S and histone 3, were amplified from host species for phylogenetic analysis. Polymerase chain reactions (PCRs) were performed using the Ex Taq PCR kit (TaKaRa, Japan). Forward and reverse primers (0.2 µM each; see electronic supplementary material, table S2) and less than 1 µg of DNA template were added to reaction mixtures. PCR products were generated by an initial denaturing step of 4 min at 94°C followed by 35 cycles at 94°C for 1 min, 55°C (50°C for COI and NADH_4_) for 1 min and 1 min at 72°C, and by a final extension at 72°C for 7 min. They were purified using Wizard SV Gels and the PCR Clean-Up System (Promega, Madison, WI). PCR products of symbiont 16S were cloned into the pCR-TOPO vectors using a TOPO TA cloning kit (Invitrogen, USA). The DNA constructs were transferred into *Escherichia coli* TOP10 cells (Invitrogen). The sequencing reaction of bacterial 16S rRNA gene clones (20–50 clones per specimen and species) and amplified eukaryotic COI, NADH4, 16S, 28S and histone 3 genes was performed using a BigDye Terminator v. 3.1 Cycle Sequencing Kit (Applied Biosystems, USA). Sequencing was performed using an ABI PRISM v. 3730 Genetic Analyzer (Applied Biosystems). Sequences were proofread using CodonCode Aligner v. 3.7.1.1 (CodonCode Corporation, www.codoncode.com). Locations of symbionts in gill tissues of *Ba. aduloides*, *Idas* nsp SAL4 and *Idas* nsp ESU R were determined using transmission electron microscopy (see electronic supplementary material, figure S1), following protocols described elsewhere [11]. Symbionts were identified by comparing their 16S rRNA sequences with GenBank databases using BLAST searches.

### (c) Model selection and reconstruction of the host tree

DNA sequences from newly obtained hosts were complemented with data from GenBank and aligned using Probalign v. 1.4 [12]. The best-fitting model of nucleotide substitution was selected for each gene and each partition within each gene using the corrected Akaike information criterion (AICc) in jModelTest v. 0.1.1 [13]. We inferred gene trees using the Bayesian method implemented in BEAST v. 1.7.2 [14] for each partitioning scheme. A Yule speciation model was used as a tree prior. We modelled rate variation among lineages using an uncorrelated lognormal relaxed clock, with the mean substitution rate fixed to 1 to get branch lengths in units of substitution per site [15]. Posterior distributions were estimated using Markov chain Monte Carlo (MCMC) sampling. Samples were drawn every 1000 steps over a total of 10 million MCMC steps. Each analysis was run four times, with mixing and convergence assessed using Tracer v. 1.5. After discarding 10% of the samples as burn-in, samples from the four runs were thinned (sampling every 4000 steps) and pooled together. The best partitioning scheme was selected for each gene by comparing marginal likelihoods using approximate Bayes factors [16] in Tracer.

We then performed a combined analysis of all five genes, using the selected partitioning scheme and substitution models. Each gene was assigned an independent relaxed clock. The protocol of the combined analysis was the same as that used for single-gene analyses, except the number of MCMC steps was increased to 20 million. The maximum-clade-credibility tree was drawn from the pooled samples. Maximum-likelihood (ML) trees were inferred using Treefinder [17], with the same partitioning scheme and substitution models as those used in the Bayesian analyses. Bootstrapping analyses (1000 replicates) were used to evaluate support for the ML tree.

### (d) Molecular dating

Three fossils were used as calibrations in the Bayesian relaxed-clock analysis and were implemented as prior distributions for ages of nodes in the tree [18].

**Figure 1.**
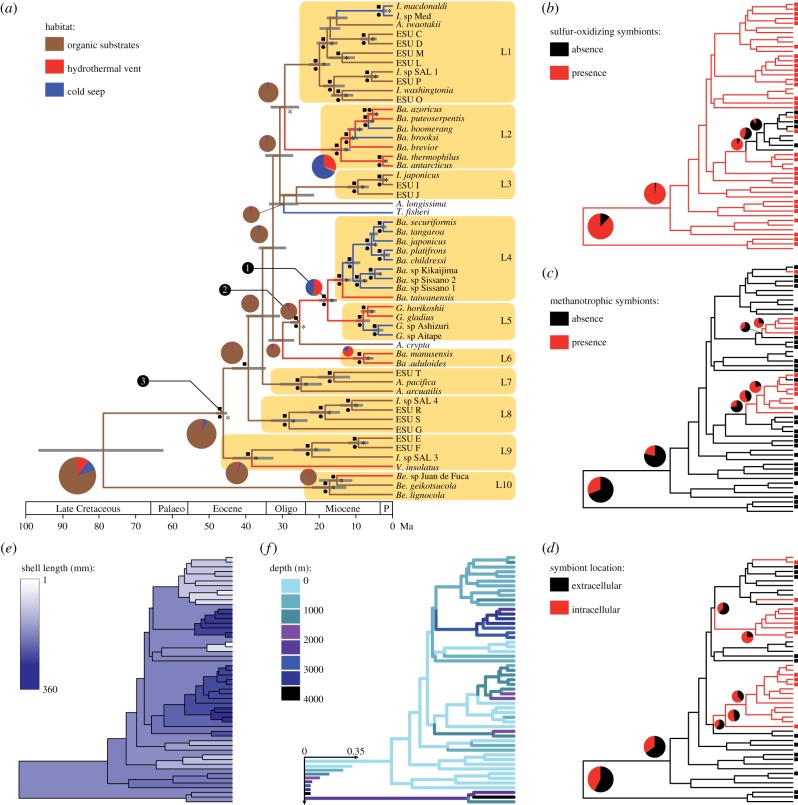
Maximum-clade-credibility Bayesian chronograms and estimates of ancestral character states. Pie charts indicate probabilities of each state at nodes discussed in the text (yellow rectangles). (*a*) Chronogram showing the evolution of habitat use. Grey bars are 95% HPD intervals of divergence time estimates. Black squares, circles and asterisks at nodes indicate posterior probabilities greater than or equal to 0.99, bootstrap values greater than or equal to 75% (95% for lineages highlighted in yellow), and nodes inferred in analyses of both nuclear and mitochondrial genes, respectively. The nodes that were assigned fossils to estimate divergence times of the ‘*childressi*’ group, and *Gigantidas* and *Vulcanidas* genera, are labelled in black circles by numbers 1, 2 and 3, respectively. Abbreviations on time scale: Palaeo, Palaeocene; Oligo, Oligocene; P, Pliocene to present; Ma, million years ago. (*b*) Evolution of the presence/absence of sulfur-oxidizing symbionts. (*c*) Evolution of the presence/absence of methanotrophic symbionts. (*d*) Evolution of the location of symbionts in the gill epithelium. Squares at tips of chronograms *b*, *c* and *d* indicate available data. (*e*) Evolution of shell lengths. (*f*) Evolution of depth use. For legibility, and because depth was discretized into nine states, a histogram was used instead of a pie chart to display probabilities at the root. Genus names are abbreviated as follows: *A.*, *Adipicola*; *Ba.*, *Bathymodiolius*; *Be.*, *Benthomodiolus*; *G.*, *Gigantidas*; *I.*, *Idas*; *M.*, *Modiolus*; *T.*, *Tamu*.

Mussels from a Middle Eocene (45 Myr) seep deposit in Washington State (USA) were recently assigned with some hesitation to *Vulcanidas* (as *Vulcanidas*? *goederti* Kiel & Amano, 2013). This hesitation stemmed from the missing data on anatomy and muscle attachment scars, while in all available shell characters *Vulcanidas*? *goederti* was more similar to *Vulcanidas* than to any other genus of deep-sea mussels [19]. Therefore, we modelled the divergence time of the clade including *Vulcanidas insolatus* Cosel & Marshall, 2010 from other species (figure 1*a*, lineage L9) using an exponential prior with a hard minimum bound of 45 Myr and a mean of 1.2 Myr. This resulted in a soft maximum matching the beginning of the Middle Eocene (48.6 Myr).

Two fossil species from seep deposits on the eastern North Island of New Zealand were recently assigned to extant clades based on muscle attachment scars. The Middle Miocene (Lillburnian; 12.7–15.1 Myr) *Gigantidas coseli* Saether *et al*. 2010 was used to calibrate the divergence of *Gigantidas* (figure 1*a*, lineage L5) from other lineages. An exponential prior with a minimum bound of 15.1 Myr and a mean of 1.3 Myr was used for this node, resulting in a soft maximum matching the beginning of the Clifdenian stage (15.9 Myr). The Early Miocene (Waitakian; 21.7–25.2 Myr) *Bathymodiolus heretaunga* Saether *et al*. 2010 was assigned to the ‘*childressi*’ clade (figure 1*a*, lineage L4 + L5) and the divergence of this clade was assigned an exponential prior with a minimum bound of 25.2 Myr and a mean of 0.7 Myr. This yielded a soft maximum matching the beginning of the Late Oligocene Duntroonian stage (27.3 Myr).

Each of the three fossil calibrations was used in a separate analysis, and then we combined them in a single analysis. For each analysis, four runs of 20 million MCMC steps, 10% burn-in and a 4000-step subsampling of individual runs were used. The COI substitution rate and the divergence between *Bathymodiolius thermophilus* Kenk & Wilson, 1985 and *Bathymodiolius antarcticus* Johnson, Won, Harvey & Vrijenhoek 2013, which is thought to have occurred at 5 Ma [20], were used to further evaluate the consistency of results obtained from our three fossil calibrations. Molecular dating was also performed using non-parametric rate smoothing in r8s v. 1.8 [21,22]. Uncertainties in the fossil calibrations were modelled using age constraints that corresponded to the intervals used in our Bayesian analyses. This analysis was run 10 times from different starting points.

### (e) Character evolution and diversification analyses

Data on habitat use, shell length, depth, presence/absence of sulfur-oxidizing and methanotrophic symbionts, and symbiont location on the gill epithelium were compiled from published sources and added to our new data (see electronic supplementary material, table S1). Because depth and depth range vary greatly among deep-sea mussels, depth was discretized using intervals of 500 m and multiple states were used to account for large depth ranges. Transitions between character states were estimated using transition matrices for categorical data [23], and a Brownian diffusion model was used for natural logarithms of shell lengths [24]. These components were added to the analysis that included all five genes and fossil calibrations, without the outgroup. As in the Bayesian dating analysis, we used four runs of 20 million MCMC steps, 10% burn-in and a 4000-step subsampling of individual runs.

To assess heterogeneity of diversification rates through time, a likelihood analysis of speciation and extinction rates [25] was conducted using the R package LASER. This analysis was based on the chronogram estimated by non-parametric rate smoothing of the ML tree (free of any speciation model). Distribution of AICc differences among a set of speciation models was obtained by simulating 5000 trees under the null hypothesis of constant diversification. The robustness of the results to sampling bias was evaluated by running simulations under the assumptions that 0, 25 and 35% of species were missing.

A similar simulation approach was carried out using the R package TESS to test whether including extinction events in the birth–death speciation model improved the likelihood score [26]. Simulations were conditioned on the age of the root and the number of species in input trees. To assess the impact of methanotrophic symbionts and intracellular symbionts on diversification rates, character-dependent Bisse [27] and Yule speciation models were fitted to the ML tree using the R package *diversitree* [28]. The significance of likelihood differences between models was tested using likelihood ratio tests. These analyses were conducted from the ML tree after removing missing data and from the patterns inferred from Bayesian analyses, assuming sampling biases of 0, 25 and 35%.

Unsupported nodes were collapsed and missing data were removed before further analyses. Correlations among categorical variables were tested (10 iterations, 1000 simulations) using Pagel's method [29] as implemented in Mesquite v. 2.75. Influences of habitat use, presence/absence of methanotrophic symbionts, and presence/absence of intracellular symbionts on natural logarithms of shell lengths and depth were tested using phylogenetic analyses of variance [30] with the R package *geiger* (5000 simulations) [31]. Correlation between natural logarithm of shell lengths and depth was tested using the phylogenetic generalized least-squares method [32] with the R package *caper*.

## 3. Results and discussion

The species tree inferred from the concatenated dataset (figure 1*a*) revealed support for 10 clades, which were consistent with those observed in previous studies [9,33,34]. Our Bayesian relaxed-clock analysis, calibrated using fossils at three nodes, yielded an estimated mean age of all deep-sea symbiotic mussels of 85 Myr (95% HPD interval: 69–102). Although this result is consistent with their divergence from other Mytilidae in the Late Mesozoic or Early Cenozoic [5,33], there is a lack of mussels at the few methane seep deposits and organic falls of this age [35–39]. This discrepancy may be due to higher levels of homoplasy along deep branches and/or to the lack of age calibrations at deep nodes in the tree. Alternatively, earliest species might be absent from the fossil record [5].

Most of the 10 extant clades started diverging from each other about 45 Ma (figure 1*a*), and the main vent and seep clades (L2, L4 and L5) diversified within the last 30 Myr, consistent with previous analyses using biogeographic calibration points [40]. The robustness of these date estimates was supported by the consistency of results obtained using each of the fossil calibrations separately (see electronic supplementary material, figure S2). Differences in mean estimates among fossil calibrations were at most 0.38% for the COI substitution rate, 0.61 Myr for the divergence between the sister species *Ba. thermophilus* and *Ba. antarcticus*, 4.05 Myr for the divergence of the genus *Gigantidas* Cosel & Marshall, 2003 (lineage L5), and 5.67 Myr for the divergence of the ‘*childressi*’ clade (lineages L4+L5). In the analysis including the three fossil calibrations, the COI substitution rate estimated across the entire tree was 1.62×10^−2^ substitutions per site per Myr (95% HPD interval: 1.22×10^−2^–2.09×10^−2^), a value higher than that estimated in vent-endemic annelid taxa, but close to those found in other invertebrates from non-chemosynthetic shallow water environments [41,42]. Our estimate for the divergence between *Ba. thermophilus* and *Ba. antarcticus* was 2.77 Myr (95% HPD interval: 1.7–4.0), a result similar to those obtained from population genetic studies [20,43].

**Figure 2.**
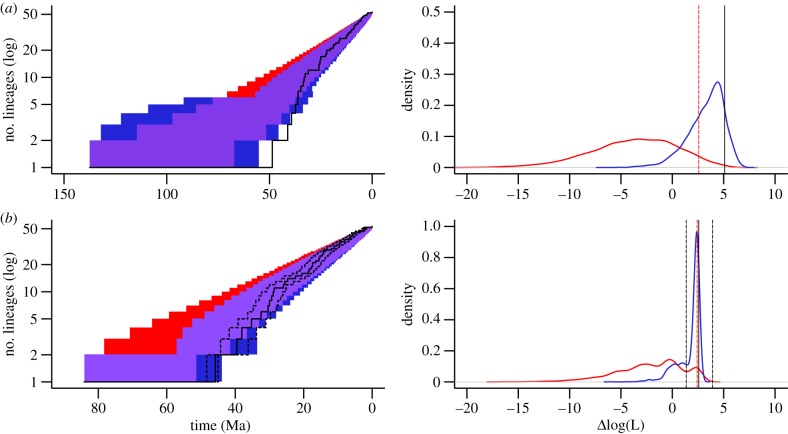
Comparison between extinction-free birth–death models (H0) and models including a mass extinction at 57 Ma (H1). Analyses were performed on chronograms inferred using (*a*) non-parametric rate smoothing and (*b*) Bayesian phylogenetic analysis. Lineages-through-time plots (left) obtained from these trees are represented by solid black lines, with additional dashed black lines for the 95% HPD interval estimated from the entire distribution of sampled Bayesian trees. Red- and blue-shaded areas correspond to 95% CIs obtained by simulation under H0 and H1, respectively, and purple areas show the overlap in confidence intervals between models. Distributions of likelihood differences between both models fitted to datasets simulated under H0 (red) and H1 (blue) are given on the right. Dashed red lines represent H0's 5% rejection levels. Likelihood differences calculated from real trees are given by solid vertical black lines, with an additional 95% HPD interval for Bayesian trees (vertical black dashed lines). Ma, million years ago; *Δ*log(L), difference between natural logarithms of likelihood.

The inference that the divergences among and within clades occurred within the last 45 Myr is remarkable because the ecological niche of deep-sea mussels—being epifaunal and taking up sulfide from the water column—was unoccupied since their inferred origin in the Late Cretaceous [44–45]. Even assuming the lower (younger) bound of the 95% HPD interval as the time of origin of the deep-sea symbiotic mussels still implies that it took 25 Myr from the origin to the diversification event seen in our tree (figure 1*a*). This long basal branch led other authors to propose the hypothesis that the genus *Benthomodiolus* (L10), which is a sister group to the remaining nine clades, may be a relic of lineages that became extinct during the global anoxia/dysoxia event associated with the Palaeocene/Eocene thermal maximum (PETM) around 57 Ma [33,46]. We found some support for that hypothesis, since the likelihood of our ML and Bayesian maximum-clade-credibility trees under a birth–death speciation model significantly improved (*p* = 0.002 and *p* = 0.024, respectively) when including an extinction event (5% survival rate) at 57 Ma. However, our simulations also show that a scenario without extinction during this time produced very similar trees, inducing a lack of statistical power in the case of Bayesian trees (figure 2). Overall, our analyses also suggested that chemosymbiotic mussels were not very diverse during the PETM and that it induced, if anything, a simple slowdown of the early diversification rather than a dramatic extinction event.

**Figure 3.**
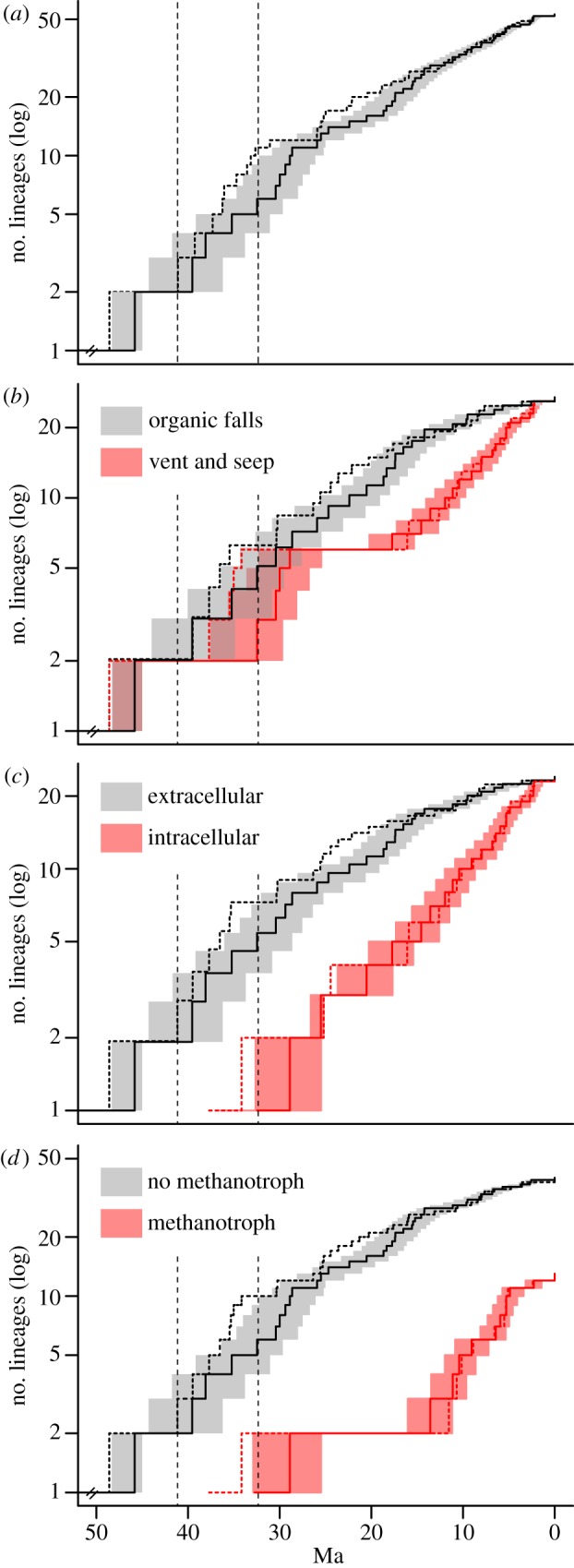
(*a*) Global and (*b*–*d*) character-dependent lineages-through-time plots (log-scaled) estimated from the ML tree smoothed using non-parametric rate smoothing (dashed lines) and from the Bayesian chronogram (solid lines). Shaded areas represent 95% HPD intervals estimated from the entire distribution of sampled Bayesian trees. Vent and seep lineages from figure 1*a* were pooled together in the habitat-dependent plot. Colour-coded groups in plots based on the location and the presence/absence of methanotrophic symbionts match the distributions of character states among taxa in figure 1*d* and 1*c*, respectively. Vertical dashed lines represent shifts of the net diversification rate estimated in the likelihood analysis of speciation and extinction rates.

The slow initial evolution of deep-sea mussels contrasts with the timing of branching events that occurred from 45 Ma. A likelihood analysis of speciation and extinction rates allowed us to identify a moderate but distinct increase in speciation rates, with about 0.17 speciation events per million years from 41 to 32 Ma (Middle Eocene to earliest Oligocene; figure 3). From 32 Ma to the present day, the estimated speciation rate was three times lower. The corresponding Yule model with three distinct speciation rates provided a significantly better fit (*p* = 0.007) to the tree than the best constant-rate model (i.e. pure birth), even in simulations assuming 25% (*p* = 0.011) and 35% (*p* = 0.009) sampling bias (table 1). Moreover, parts of the gene trees and species tree that fall within that interval are consistently characterized by poorly resolved nodes and short internode distances (figure 1*a*). It is worth noting that a complementary analysis of speciation rates from 41 Myr to the present suggests that a slow decrease of speciation rates, following a Weibull distribution (*β* = 1.42), might also be an alternative to the simple Yule model (*χ*^2^ = 8.33, *p* = 0.004). This indicates that mussels have continued to diversify after their initial radiation, as corroborated by the existence of several young species and species complexes [20,46].

**Table 1.** Results of the likelihood analysis of speciation and extinction rates (LASER). Characteristics of each model are abbreviated as follows: RC, rate constant; RV, rate variable; L, likelihood; *r*1, first diversification rate; *r*2, second diversification rate; *r*3, third diversification rate; *a*, extinction fraction of the birth–death model (ratio extinction/speciation); *xp*, *x*-parameter from the exponential variant of the density-dependent speciation rate (DDX) model; *k*, *k*-parameter from the logistic variant of the density-dependent speciation rate (DDL) model; *s*1, first break in diversification rate (million years); *s*2, second break in diversification rate (million years); dAIC, the difference in AIC scores between the model and the overall best-fit model.

model	parameters	type	L	*r*1	*r*2	*a*	*k*	*xp*	*s*1	*s*2	*r*3	AIC	dAIC
pure birth	*r*1	RC	−54.49	0.05	n.a.	n.a.	n.a.	n.a.	n.a.	n.a.	n.a.	111.0	12.4
birth–death	*r*1, *a*	RC	−54.44	0.04	n.a.	0.02	n.a.	n.a.	n.a.	n.a.	n.a.	112.9	14.3
DDL	*r*1, *k*	RV	−54.45	0.05	n.a.	n.a.	429	n.a.	n.a.	n.a.	n.a.	112.9	14.3
DDX	*r*1, *x*	RV	−54.03	0.03	n.a.	n.a.	n.a.	−0.13	n.a.	n.a.	n.a.	112.1	13.5
Yule 2 rates	*r*1, *r*2, ts	RV	−50.30	0.01	0.05	n.a.	n.a.	n.a.	39.6	n.a.	n.a.	106.6	8.0
Yule 3 rates	*r*1, *r*2, *r*3, *s*1, *s*2	RV	−44.29	0.01	0.17	n.a.	n.a.	n.a.	41.2	33.8	0.05	98.6	0

To test if and how symbiont type and location have influenced the diversification of mussels, we estimated the ancestral states of these characters. Our results indicate that sulfur-oxidizing symbionts are an early acquisition going back to the stem of the group, almost 30 Myr before the main burst of diversification (figure 1*b*). In contrast, the ability to host intracellular symbionts was detected in our tree from the Late Oligocene onward, and methanotrophic symbionts only from the Middle Miocene onward (figures 1*c*,*d* and 3). This suggests that the evolution of the various symbiotic relationships did not trigger the Middle Eocene–Early Oligocene burst of diversification, although the association with sulfur-oxidizing symbionts was probably a prerequisite. A potential environmental trigger for this burst might have been the appearance of whales, because their carcasses, which produce large amounts of sulfide, are thought to be dispersal stepping stones [47]. This hypothesis was originally based on the congruence of molecular age estimates for the chemosymbiotic bivalve family Vesicomyidae and the rise of whales, but it has been challenged because Eocene and Oligocene whale falls lacked associated vesicomyid fossils [48]. However, these early whale falls were abundantly colonized by mussels, and thus the ‘whale stepping stone’ hypothesis might apply to mussels. Alternatively, the geological record shows a rapid increase in the abundance of seep carbonates in the Late Eocene [49], indicating a worldwide increase in methane seepage. Analogous to the rationale of the ‘whale stepping stone’ hypothesis, this increase in habitat availability and in potential dispersal stepping stones could have triggered the Late Eocene radiation. A third possible trigger is a drop in deep-water temperature beginning in the Middle to Late Eocene, associated with the initial glaciation of Antarctica [50]. Low ambient water temperatures could have decreased the metabolic rate of mussel larvae, thus increasing their longevity and enhancing their dispersal capability. This would have been an advantage for mussels living in patchy deep-sea habitats such as vents, seeps and organic substrates.

Even if the acquisition of intracellular and methanotrophic symbionts was not involved in the Middle Eocene to Early Oligocene radiation, symbionts played a role in the subsequent evolution of the group. Pagel's tests showed that both the presence of methanotrophic symbionts and the intracellular location of symbionts are correlated with the occurrence at vents and seeps (table 2). This supports the adaptive significance of these two character states and the hypothesis that symbionts ‘increase the metabolic capabilities, and therefore the number of ecological niches’ colonized by mussels [51, p. 475]. We further explored this hypothesis by comparing simple Yule speciation models with models in which the diversification rates depend on character states. Even when assuming high levels of sampling bias, the results suggest that diversification rates are higher in lineages hosting intracellular symbionts and methanotrophic symbionts (table 3). The acquisition of these two characters is correlated with a second wave of mussel diversification at vents and seeps (figure 3), and is probably the key to the success of vent and seep clades (lineages L2, L4–L6 in figure 1*a*). These include nearly as many species as from organic falls, but have higher speciation rates because they started diversifying only since the Miocene. Methanotrophic symbionts were only acquired in species hosting intracellular symbionts (figure 1*c*,*d*; electronic supplementary material, table S1) and both characters are strongly correlated (table 2). This suggests that the ability to host intracellular symbionts was a prerequisite for the acquisition of methanotrophic symbionts.

**Table 2.** Tests of correlations between characters. Model used, number of species for which data were available, criteria estimated, degrees of freedom (d.f.) and *p*-values are given for each pair of characters.

model	variables	species	criterion	d.f.	*p*-value
Pagel's test	methanotroph ∼ symbiont location	24	*Δ*log(L) = 4.916		0.001
	methanotroph ∼ habitat	36	*Δ*log(L) = 3.900		0.024
	symbiont location ∼ habitat	24	*Δ*log(L) = 4.331		0.006
pGLS	log(size) ∼ depth	48	intercept: *t* = 4.460		0
			slope: *t* = 0.471		0.640
			*F* = 0.222	46	0.802
pAnova	log(size) ∼ habitat	48	*F* = 61.328	1	0
			phylogenetic *p*-value		0
	log(size) ∼ symbiont location	24	*F* = 9.571	1	0.005
			phylogenetic *p*-value		0.053
	log(size) ∼ methanotroph	36	*F* = 3.341	1	0.057
			phylogenetic *p*-value		0.143
	log(depth) ∼ symbiont location	24	*F* = 6.994	1	0.015
			phylogenetic *p*-value		0.097
	log(depth) ∼ methanotroph	36	*F* = 6.153	1	0.018
			phylogenetic *p*-value		0.067

**Table 3.** Impact of methanotrophic symbionts and symbiont location on diversification rates. Character-dependent Bisse (H1: diversification rate *λ*_0_ under state 0 and *λ*_1_ under state 1) and simple Yule (H0: global diversification rate *λ*_Yule_) speciation models were fitted to sampled data and patterns inferred during the Bayesian analysis. Levels of sampling bias considered are expressed as ratios and reflect estimated proportions of extant species with state 0 and 1 included in the phylogeny. Likelihood ratios (LRs) calculated between Bisse and Yule models and associated *p*-values (*p*) are given.

dataset	methanotrophic symbionts					symbiont location				
	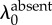	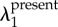	*λ*_Yule_	LR	*p*-value	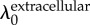	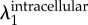	*λ*_Yule_	LR	*p*-value
sampled data	0.010	0.088	0.037	12.938	0	0.004	0.055	0.028	10.276	0.001
sampled data, bias ∼ 0.75 : 1	0.012	0.099	0.041	12.517	0	0.003	0.064	0.031	9.891	0.002
sampled data, bias ∼ 0.65 : 1	0.013	0.105	0.043	12.346	0	0.003	0.068	0.033	9.749	0.002
sampled data, bias ∼ 1 : 0.75	0.039	0.010	0.091	14.312	0	0.007	0.057	0.031	11.562	0.001
sampled data, bias ∼ 1 : 0.65	0.039	0.010	0.093	14.880	0	0.007	0.060	0.032	12.303	0
inferred pattern	0.022	0.099	0.043	14.711	0	0.021	0.084	0.043	8.146	0.004
inferred pattern, bias ∼ 0.75 : 1	0.027	0.107	0.049	11.871	0.001	0.038	0.084	0.047	6.948	0.008
inferred pattern, bias ∼ 0.65 : 1	0.030	0.112	0.052	10.562	0.001	0.041	0.084	0.050	5.928	0.015
inferred pattern, bias ∼ 1 : 0.75	0.022	0.105	0.045	17.79	0	0.021	0.092	0.046	12.515	0
inferred pattern, bias ∼ 1 : 0.65	0.022	0.107	0.045	19.315	0	0.033	0.110	0.047	16.696	0

Our estimation of ancestral habitat types indicates that vents and seeps were colonized from organic falls independently in six lineages, consistent with the hypothesis that these mussels took ‘wooden steps to deep-sea vents’ [8]. These habitat transitions were unidirectional, suggesting that the adaptation to vents and seeps is an irreversible specialization. This might be partly explained by the evolution of symbioses toward more integrated relationships (i.e. intracellular symbioses) and the concomitant reduction of the digestive tract in several species [46,52]. Specialization might also have resulted from the evolution of other anatomical and physiological traits.

Indeed, we found a significant correlation between habitat type and shell size (table 2). We also inferred a trend towards larger shells in vent/seep species from the Early to Middle Miocene onwards and towards smaller shells in lineages living on organic falls (figure 1*e*). This pattern is remarkably consistent with the fossil record, which shows that seep-inhabiting mussels did not exceed 50 mm in length until the Early Miocene [53–55], and then rapidly increased in size from 100 mm in the Middle Miocene to more than 300 mm today. Compared with the typically small and ephemeral organic substrates on the seafloor, vents and seeps provide large amounts of energy and habitable space that might explain the increased size of species living there [54]. By contrast, species living on sparse organic debris would benefit from becoming smaller to reduce competition for space and/or for early allocation of resources to reproduction. The latter hypothesis is corroborated by studies suggesting that mussels from organic substrates reach sexual maturity quickly and at much smaller size [56–58] than their vent and seep relatives [59].

Earlier studies indicated a trend of successive adaptation to greater water depth among mussels [33]. This trend is also seen in our data for post-Eocene taxa (figure 1*f*); however, it coincides with habitat transitions from organic substrates to vents and seeps, and the latter habitats typically occur in deeper water than organic falls (Mann–Whitney: *p* = 0.001). Thus, the colonization of greater depth might just be a consequence of the habitat transitions. Alternatively, there may be a systematic sampling bias because wood and bones are much more difficult to locate on the vast deep-sea floor than vents and seeps, which can be detected using temperature anomalies or seismically reflective sediment layers [60,61].

In conclusion, the evolutionary history of chemosymbiotic deep-sea mussels shows all the characteristics of an adaptive radiation: a burst of diversification into a suite of new habitats due to an ecological opportunity, along with physiological and morphological adaptations to these habitats. Although these mussels rely on their symbionts for nutrition, it is remarkable that the initial acquisition of sulfur-oxidizing symbionts did not trigger a major radiation. This adds to the growing evidence that an early burst might be rare in adaptive radiations [62]. The link of the burst of diversification in the Middle Eocene to Early Oligocene to ecological opportunities such as increased habitat availability or dispersal capability raises the question of whether other chemosymbiotic deep-sea taxa were similarly affected. Interestingly, a compilation and reassessment of molecular age estimates for 14 vent/seep taxa showed that the inferred origins of six of these (Alvinellidae, Lepetodrilidae, *Alviniconcha*/*Ifremeria*, *Provanna*, Bresiliidae and Bythograeidae) fall within the time interval of the mussels’ burst of diversification [41]. For the mussels, we consider the acquisition of sulfur-oxidizing symbionts as a prerequisite for their adaptation to, and successful radiation within, chemosynthetic environments. By contrast, the subsequent acquisition of methanotrophic symbionts allowed the colonization of new niches within the vent and seep environment, and resulted in a second wave of diversification.

## Data accessibility

Mussel sequences were deposited in Genbank under accession numbers HF545021–HF545099. Symbiont sequences were deposited under accession numbers KF657320–KF657326. The Beast xml file including the full dataset is available as an electronic supplementary file.
